# Fast and Scalable Sample Preparation for Integrated Metabolome and Exposome-Wide Association Studies (MWAS/ExWAS)

**DOI:** 10.1021/acs.analchem.6c03820

**Published:** 2026-08-23

**Authors:** Julia Füreder, Amira Müller, Giulia Guerra, Sabina Sieri, Max Lennart Feuerstein, Benedikt Warth

**Affiliations:** † Department of Food Chemistry and Toxicology, Faculty of Chemistry, 27258 University of Vienna, 1090 Vienna, Austria; ‡ Vienna Doctoral School in Chemistry (DoSChem), Faculty of Chemistry, University of Vienna, 1090 Vienna, Austria; § Epidemiology and Prevention Unit, 9329 Fondazione IRCCS Istituto Nazionale dei Tumori, 20133 Milan, Italy; ∥ Exposome Austria, Research Infrastructure and National EIRENE Node, 1090 Vienna, Austria

## Abstract

Broad coverage exposome- and metabolome-wide association studies rely on advanced instrumentation, typically anchored in liquid chromatography–high-resolution mass spectrometry (LC–HRMS) to investigate exposure-effect associations. Sample preparation for biological fluids is a delicate matter, as it is essential to strike a pragmatic balance between sensitivity, chemical coverage, robustness, and time efficiency to meet the requirements of large-scale epidemiological studies. Here, we established a protein precipitation workflow in a scalable, high-throughput format for human plasma and urine. Different protocols were evaluated utilizing a xenobiotic mixture containing >200 exposure compounds based on extraction recovery, repeatability, and applicability for nontargeted analysis (NTA) and suspect screening. The tested high-throughput options included a protein precipitation workflow in 96-well plates and a phospholipid removal plate. For urine, a dilute-and-shoot approach was additionally tested. The plate-based protein precipitation resulted in superior performance with acceptable extraction recoveries (*R*_E_, 60–140%) for >70% of the highly diverse analyte panel in plasma and >80% in urine, and acceptable repeatability with relative standard deviations of <30% for more than 95% of analytes in plasma and 80% in urine. NTA results exhibited only a small number of altered annotated features compared to a tube-based protocol used for benchmarking, while increasing the overall time efficiency by a factor of five. To further test the protocol, it was applied to the NIST plasma standard reference material SRM1950. A total of 22 toxicants were identified and (semi-)­quantified, with most concentrations comparable to available reference values. The results demonstrate the suitability of the established protocol for combined MWAS/ExWAS.

## Graphical abstract

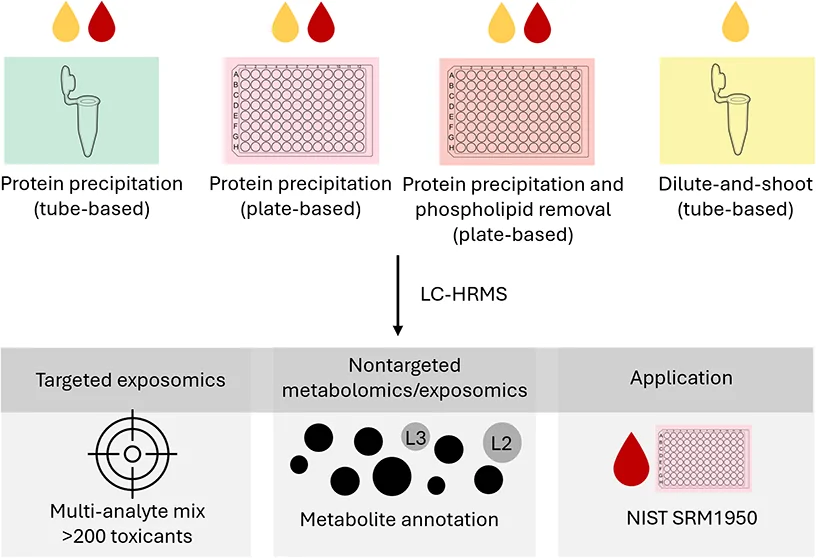

Throughout their lifetime, humans are constantly exposed to a multitude of dietary and environmental chemicals that stem from various exposure sources such as diet, personal care products, lifestyle choices (e.g., smoking), or environmental pollution.^1,2^ Positive associations between specific chemical exposure classes, e.g., per- and polyfluoroalkyl substances (PFAS) or endocrine disrupting chemicals (EDCs), and disease outcomes such as thyroid- or breast cancer have been reported but typically remain elusive and hard to replicate.^3,4^ Furthermore, it is crucial to consider potential mixture effects of chemicals on health and disease outcomes.^5^ The integrated compilation of all chemical, biological, physical, and psychosocial influences that impact biology is encompassed by the term “exposome”.^2^ Recent studies increasingly focus on the systematic discovery of relationships between exposures and phenotypes, referred to as exposome-wide association studies (ExWAS).^6^ To bridge the gap between environmental exposures and disease outcomes, inclusion of endogenous mediators is pivotal and often encompassed in the definition of the exposome.^1,7^ The endogenous counterpart that should be integrated with ExWAS for combining exposure and effect measurements is typically referred to as a metabolome-wide association study (MWAS). Several practical challenges must be overcome to generate useful and comprehensive data for integrated MWAS/ExWAS from an analytical perspective. These include coverage of a vast number of analytes with diverse physicochemical properties representing a broad chemical space,^8^ and concentrations spanning a wide dynamic range.^7^ In addition, high sample throughput and method reproducibility are required to achieve sufficient data quality and statistical power.^8^ Mass spectrometry has emerged as the key technology used to broadly assess environmental exposures and endogenous metabolites in biological matrices.^9,10^ Nontargeted analysis based on high-resolution mass spectrometry (HRMS) enables the potential discovery of unknown compounds.^11^ Furthermore, HRMS coupled to front-end separation techniques such as liquid chromatography (LC) offers the possibility of retrospective screening for contaminants of emerging concern.^11–13^ For targeted as well as nontargeted exposomics, the sample preparation of complex biological matrices remains challenging and is an important strategic decision.^14^ Requirements for ExWAS sample preparation are broad chemical coverage without systematic discrimination of specific analyte classes, sufficient sensitivity for trace analysis, reduction of sample complexity, method reproducibility and robustness, and a balance between time and resource efficiency.^8,14^ In exposomics and metabolomics studies on biological fluids, liquid–liquid extraction protocols, as well as dilute-and-shoot (DNS) approaches, are considered promising due to their nondiscriminatory properties.^14–17^ Simple DNS offers the highest sample throughput capabilities, at the cost of reduced method sensitivity due to higher dilution factors, which limits its applicability for exposomics.^18^

The aim of this study was to establish a robust and balanced high-throughput alternative to a validated tube-based protein precipitation protocol (PPT) for urine and plasma^16,17,19^ and to demonstrate its application for integrated exposomics and metabolomics. Three scalable protocols were tested: a PPT workflow in 96-well plates, a 96-well plate-based phospholipid removal workflow (PLR) for urine and plasma, and a DNS workflow for urine only. Method performance was compared to the benchmark method in terms of extraction recovery and repeatability, and its applicability for nontargeted metabolomics/exposomics was evaluated by means of chemical coverage. The most promising protocol was applied to a certified reference material (SRM1950) to compare the method performance with previously published studies in the field.

## Experimental Section

### Chemicals and Biological Samples

A mixture of 223 authentic reference standards was spiked into pure solvent, pooled plasma, and pooled urine to evaluate sample preparation performance. The targeted analytes include various toxicants and other xenobiotics in terms of chemical classes, exposure routes, and physicochemical properties and were based on previous work.^20^ More information, including chemical formula, identifiers, log *P* values, and calibration levels, can be found in the Supporting Information and Table S1.

Commercially available pooled human Li-heparin plasma was purchased from Innovative Research, Inc. (IPLAWBLIH-31982, Novi, Michigan, United States). Standard reference material 1950 was obtained from the National Institute of Standards and Technology (NIST, Gaithersburg, Maryland, United States). A pooled urine sample was obtained from a female volunteer who avoided foodstuff and beverages stored in plastic containers, foods rich in phytoestrogens, and cosmetics containing parabens for 2 days prior to sample collection.^19^ Stocks, working solutions, and urine samples were stored at −20 °C, and plasma samples were stored at −80 °C.

### Sample Preparation

#### Experimental Design

For each biological matrix and sample preparation approach, nonspiked quality control (QC) samples were spiked with a mixture containing 223 xenobiotics before (pre-extraction-spike) and after (post-extraction-spike) extraction at a low concentration level (calibration level 3 from Table S1) and a high concentration level (calibration level 30). Pre-extraction-spiked and nonspiked QC samples were prepared in triplicates. Process blanks were prepared in triplicate by replacing the sample matrix with an equal volume of water. Furthermore, seven-point solvent and six-point matrix-matched calibration curves (post-extraction spikes) were prepared (Figure 1).

**Figure 1:**
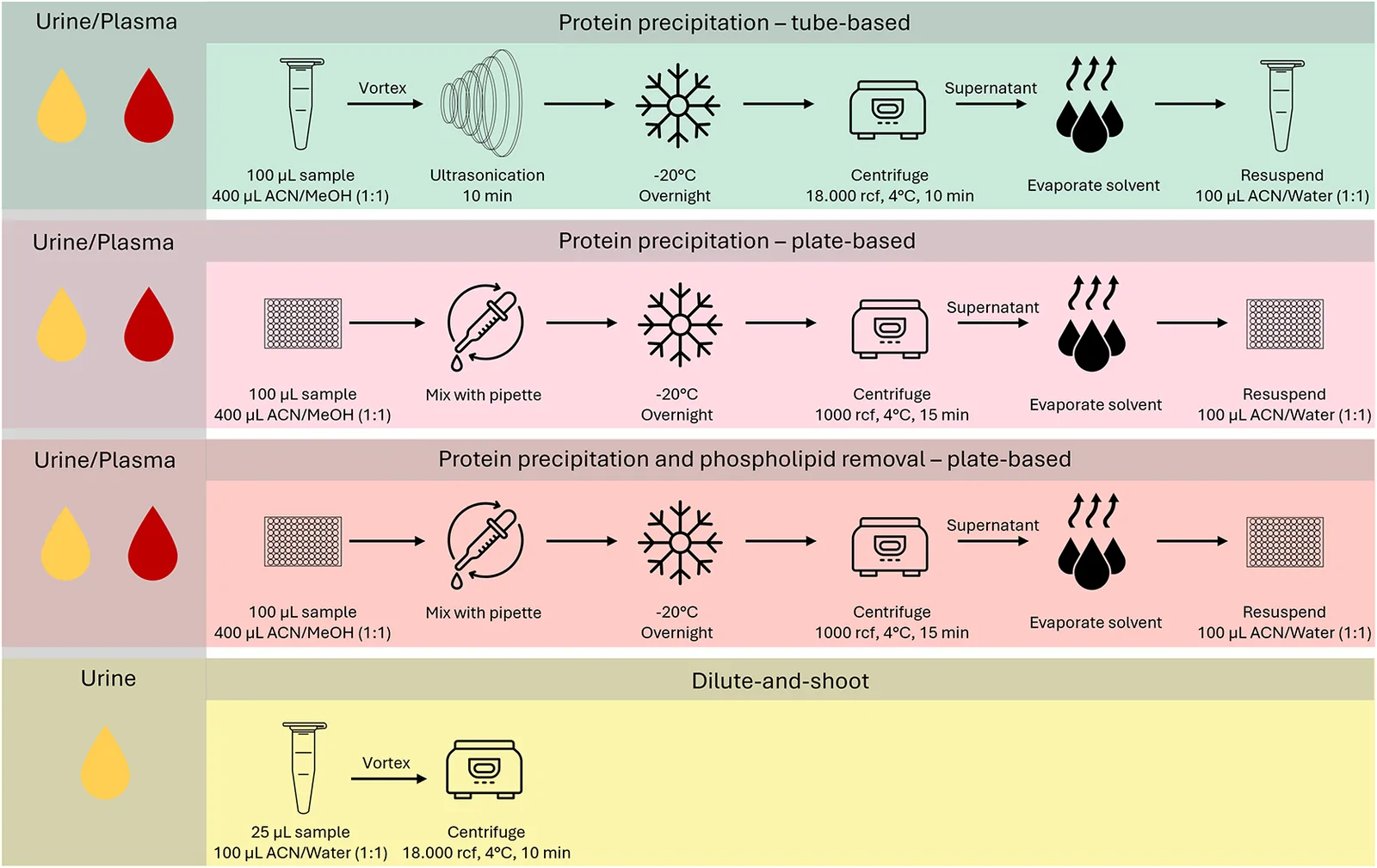
Overview of (A) experimental design, (B) the four tested sample preparation protocols, and (C) the data acquisition sequence outline for LC–HRMS measurements. (A) Blank samples were prepared to account for background contamination in each workflow. Nonspiked, pre-extraction-spiked, and post-extraction-spiked quality control (QC) samples were measured to determine extraction recoveries and repeatability of extraction procedures for >200 exposure compounds at two concentration levels. Matrix-matched calibration curves were prepared for compound quantification. (C) LC–HRMS data was acquired in full scan mode with fast polarity switching in the depicted sequence design. Each workflow started with the injection of process blank samples, followed by non- and pre-extraction-spiked samples, matrix-matched, and external calibration divided by repeat injections of the process blanks. Samples with gray shadings were used for instrument equilibration and thus were not included during data analysis.

#### Sample Preparation Protocols

Since the PPT-plate-based workflow eventually showed the best performance, this protocol is described here in detail. PPT-tube-based and PLR (ISOLUTE PLD+, Biotage, Uppsala, Sweden) workflows were prepared similarly. A detailed description of the other sample preparation protocols, including the DNS workflow, is reported in the Supporting Information and Figure 1. During the whole procedure of the PPT-plate-based workflow, samples were kept on ice. In short, 100 μL of sample was mixed with 400 μL of extraction solvent [acetonitrile (ACN)/methanol (1:1, v/v)] in a 96-well filter plate (ISOLUTE PPT+, Biotage, Uppsala, Sweden). For pre-extraction-spikes, 300 μL extraction solvent was mixed with 100 μL of the standard mixture prepared in extraction solvent. Then, proteins were precipitated at −20 °C overnight, and centrifugation was performed at 1000 rcf and 4 °C (15 min) to separate the protein pellet from the supernatant. Samples were evaporated to dryness in a vacuum concentrator at 7 °C (CentriVap, Labconco, Kansas City, Missouri, United States) and stored at −20 °C until resuspension. Residues were reconstituted in 100 μL of reconstitution solvent (ACN/water (1:1, v/v)) and mixed for 10 min on a plate shaker (Incu-Mixer MP4, Benchmark Scientific, Sayreville, New Jersey, United States) at 1000 rpm and room temperature. Samples for matrix-matched calibration were reconstituted in 100 μL of the standard mixture with the respective concentration prepared in ACN/water (1:1, v/v). Finally, the extracts were transferred to LC vials. Plasma samples were measured directly, and urine samples were stored at −20 °C until analysis.

### Data Acquisition

Samples were analyzed using a Vanquish Horizon LC coupled to an Orbitrap Exploris 480 (Thermo Fisher Scientific) equipped with a heated electrospray ionization source (H-ESI). The LC method utilized an Acquity HSS T3 column (1.8 μm, 2.1 × 100 mm, Waters). Mobile phase A was composed of water with 0.3 mM ammonium fluoride as an additive, while mobile phase B was 98% (v/v) ACN with 2% water. The column oven and autosampler temperatures were set to 40 and 7 °C, respectively. The flow rate was 0.6 mL min^–1^, and the injection volume was 5 μL. The 15 min gradient started with 5% eluent B, which was held for 0.5 min and then raised linearly to 100% B within 9 min. This was held for 3 min and then decreased to 5% for a 3 min re-equilibration step. The MS source parameters were set as follows: ion transfer tube temperature 280 °C, vaporizer temperature 400 °C, sheath gas 50 arb, aux gas 10 arb, and sweep gas 2 arb. The ion capillary voltage was set to 3000 V in positive mode and 2500 V in negative mode. Full scan data was acquired in fast polarity switching mode (sequence design described in Figure 1C). Full scan parameters were set as follows: resolution 90,000 at *m*/*z* 200, scan range *m*/*z* 60–900, and AGC target 10^6^. The sequence was designed to include samples for instrument equilibration at the beginning of the measurement and after switching from solvent to matrix (or vice versa). These equilibration injections were not included in the data evaluation (gray shadings in Figure 1C). For identity confirmation, fragment spectra were generated using data-dependent acquisition (DDA) mode, and data was acquired separately for each polarity. To ensure maximum coverage, iterative inclusion and exclusion lists were created using the AcquireX vendor workflow. DDA MS2 parameters were as follows: resolution 60,000 at *m*/*z* 200, HCD collision energies 20% and 40%, and 80% normalized collision energy. SRM1950 samples were measured with the same instrument configuration as described above following minor modifications: Mobile phase B was composed of 95% (v/v) ACN with 5% water and the gradient was prolonged to a total time of 20 min by extending the re-equilibration step from 3 to 8 min.

Several types of QC samples were used to ensure adequate data quality. This includes solvent and process blanks to account for background contamination, non-, pre-, and post-extraction-spiked QC samples to evaluate protocol performance, as well as matrix-matched calibration and external calibration in neat solvent. A system suitability test was performed before the start of each sequence (Supporting Information and Table S2). Details of the LC–HRMS method for SRM1950 and the complete QC protocol are available in the Supporting Information.

### Data Analysis

#### Targeted Exposomics Evaluation of >200 Exposure Compounds

Peak integration for targeted data evaluation was performed using Skyline (version 25.1.0.237). Further data processing was done in R Studio (version 2022.12.0 + 353) and Microsoft Office 365. Workflows were compared based on the regression coefficient (*R* ^2^) of the calibration curves in matrix, the lowest spiking level within the linear range of the calibration curve (LLOQ), extraction recovery (*R*_E_), and repeatability of the extraction procedure for two spiking levels each. Calibration curves (1/*x*-weighted) for standards in matrix were generated, and multipoint calibration curves (*n* ≥ 2) with regression coefficients of at least 0.9 were considered for further data evaluation. Extraction recovery (*R*_E_) was calculated in triplicate for low- and high-spiking levels as the ratio of peak areas in pre-extraction and post-extraction-spiked samples after subtracting the average peak area in nonspiked samples. If *R*_E_ could not be determined, e.g., due to high background concentrations in matrix or nondetected peaks in pre- and/or post-extraction-spiked samples, semiquantitative data evaluation was performed without considering *R*_E_. For analyte quantification, maximum peak areas in process blanks were subtracted from peak areas in samples and QCs, including matrix-matched calibration. Compounds in nonspiked samples were quantified using standard addition, i.e., by dividing the intercepts by the slopes of the matrix-matched calibration curves. Resulting concentrations were corrected for *R*_E_ and, in the case of DNS, for the dilution factor. Analytes in NIST SRM1950 were quantified using matrix-matched calibration, and concentrations were corrected for *R*_E_. If compounds were present in the nonspiked blank matrix, intercepts were set to zero, and process blank-corrected peak areas of the SRM1950 were divided by the slope and corrected for *R*_E_. Analytes for which extraction recovery could not be assessed, calibration curve showed an insufficient number of data points (less than 5), or where the peak area was outside the calibration curve range, were classified as semiquantitative. Repeatability of the extraction procedure was determined as relative standard deviations (RSD) of extraction recoveries in triplicate pre-extraction-spiked samples.

#### Nontargeted Metabolomics/Exposomics

Untargeted data analysis including peak detection, alignment, and compound annotation was performed using MS-DIAL (4.9.221218). MS1 and MS2 data were extracted with mass tolerance of 0.0025 Da for MS1 and 0.01 Da for MS2, and an intensity cutoff of 100,000 was used during data extraction. MassBank of North America (MoNA) was used for annotation of spectral matches. MS-DIAL settings are reported in detail in Table S3. Annotations were curated manually and only annotations with mass error <5 ppm were accepted. The annotations were classified based on level of confidence^21^ as follows: “Level 1” identifications were confirmed by reference standards, “Level 2” annotations were based on spectral library matches, whereas “Level 3” annotations resulted from spectral library matches with the possibility of isomeric structures. Further data processing and visualization were done using *R* Studio (version 2022.12.0 + 353), MetaboAnalyst (version 6.0), and Microsoft Office 365. To allow comparisons between data sets, peak areas of the DNS data set were multiplied with the corresponding dilution factor. Feature tables were filtered based on process blank levels and repeatability of QC injections: average peak areas in samples had to be three times higher than average peak areas in process blanks, and the RSDs in replicate injections had to be <30%.^22^ Feature tables from positive and negative mode were merged, peak intensities were log-transformed (base 10) using MetaboAnalyst (version 6.0),^23^ and autoscaling was applied. Fold change analysis with a fold-change threshold of 2 was applied to compare the suitability of all protocols for nontargeted small molecule analysis. Further data processing details can be found in the Supporting Information.

## Results and Discussion

### Performance Evaluation for >200 Exposure Compounds

Given the vast number of targeted chemicals in exposomics, it is crucial to find a pragmatic balance between sensitivity, chemical coverage, and robustness to address comprehensive biological questions.^14^ Still, it is important to consider that many toxicants are present at very low concentrations (e.g., <0.01 ng/mL) in real-life samples.^20^ Therefore, two spiking levels in the ng/mL and sub ng/mL concentration range were used to spike the QC samples. These spiking levels were intended to mimic real-life concentrations of toxicants in biofluids and to explore the limits of the tested protocols.

### Lower Limit of Quantification

Median LLOQs for plasma were 0.8 ng/mL, 0.8 ng/mL, and 0.5 ng/mL for the PPT-tube, PPT-plate, and PLR workflows, respectively. For urine, the median LLOQs were 0.9 ng/mL for the PPT-tube-based workflow and 0.6 ng/mL for the others. *R* ^2^, slope, intercept, and LLOQ for each analyte are listed in Tables S4 and S5 for plasma and urine, respectively. Figure S1 exemplarily shows the matrix-matched calibration curve for the mycotoxin zearalenone in urine across the four tested workflows. Apart from matrix-matched calibration, seven-point solvent calibrations were repeatedly injected throughout the batch (Figure 1C). For some analytes, we observed signal drifts in solvent standards throughout the batch and better signal stability in spiked sample matrices (Table S6).

### Extraction Recovery

Extraction recovery (*R*_E_) was assessed for two spiking levels per matrix and protocol (Figure 2A). In total, *R*_E_ could be calculated for roughly two-thirds of the exposure compounds at the high-spiking level, and for approximately one-third of compounds at the low-spiking level, depending on the combination of workflow and matrix. Clearly, a lower number of compounds were detected at lower spiking concentrations due to the limited sensitivity of HRMS and the intention to spike low levels at the edge of the method’s detection limit. To classify method performance, an *R*_E_ between 60% and 140% was deemed acceptable for the chemically diverse multianalyte mix.^22^ In plasma, the PPT-tube-based workflow showed superior results for both spiking levels, with a median *R*_E_ of 86% for the high-spiking level and 93% for the low-spiking level, with 82% and 89% of compounds within the acceptable range. The PPT-plate-based workflow had a median *R*_E_ of 83% for the high-spiking level (78% within 60–140%) and a median *R*_E_ of 78% for the low spiking level (73% within 60–140%). Substantially lower *R*_E_ were observed for the PLR approach, with median *R*_E_ of 48% and 56% for high- and low-spiking levels, and 10% and 28% within the acceptable range, respectively. Compared to a hybrid solid-phase extraction and phospholipid removal workflow previously reported by Sdougkou et al., 2023,^24^ this study reports *R*_E_ of 81 ± 7%, 87 ± 3%, and 56 ± 23% (PPT-tube-based, PPT-plate-based, and PLR) for a concentration of 5 ng/mL of enterolactone, while the former study reports 83 ± 9% for the same concentration. For atrazine, *R*_E_ of 69 ± 1%, 60 ± 4%, and 38 ± 12% for 9 ng/mL were reported in this study, while 83 ± 5% were reported previously for 5 ng/mL. For some compounds (e.g., cotinine or ibuprofen), *R*_E_ could not be reported, since concentrations in the nonspiked plasma sample were too high to allow matrix-matched calibration. This issue may be overcome using isotopically labeled internal standards in the future, or by the analysis of multiple dilutions of the samples, but would result in increased analysis costs. In urine, the PPT-tube-based workflow showed the highest median *R*_E_ of 112% for the high-spiking level and 71% for the low-spiking level, with 87% and 71% of compounds within the acceptable range of 60–140%. The PPT-plate-based workflow is similar to the median *R*_E_ of 101% and 120%, with 90% and 83% acceptable. The PLR workflow shows lower extraction recoveries than the other workflows, with median values of 57% and 67%, respectively. Only 41% for the high-spiking level and 73% for the low-spiking level show acceptable *R*_E_. In one of our previous studies, a similar tube-based approach with minor modifications was used, and comparable results were obtained for identical spiking levels.^25^ Using our previous method, *R*_E_ for trimethoprim was 92 ± 2% and 98 ± 2% for low and high-spiking levels, respectively, compared to 97 ± 3% and 115 ± 2% reported herein. For florfenicol, *R*_E_ was 110 ± 7% and 114 ± 3% for low- and high-spiking levels, respectively, while we report 67 ± 12% for low and 104 ± 8% in this study. Overall, results indicate the suitability of both PPT approaches for highly diverse multianalyte panels in urine and plasma, while direct translation to PLR-plates without further optimization resulted in substantially lower *R*_E_. Summarized results for each protocol and matrix can be found in Tables S4 and S5 for plasma and urine, respectively.

**Figure 2:**
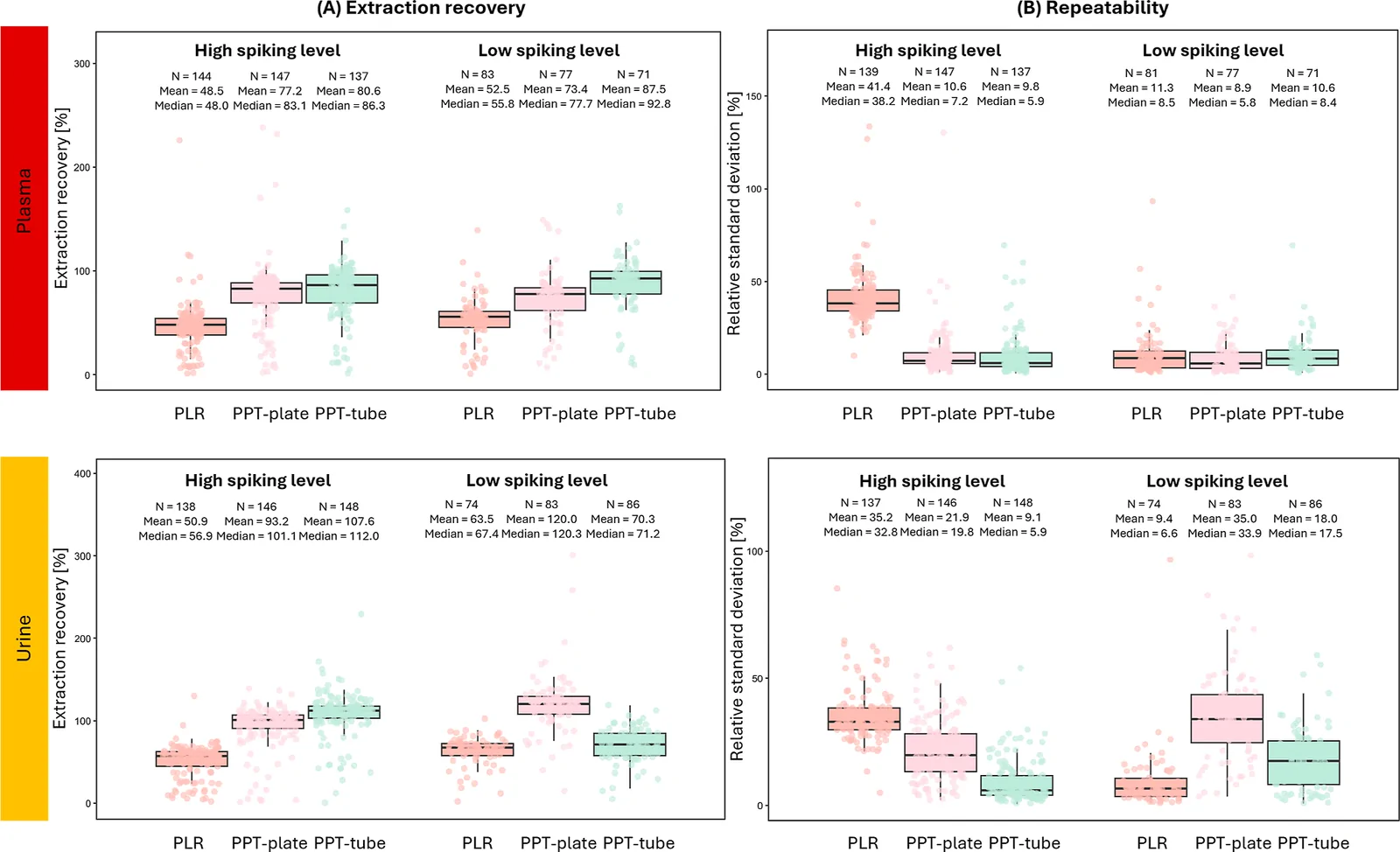
Evaluation of the method performance of three different extraction protocols for the targeted analysis of >200 exposure compounds, including (A) extraction recoveries for two spiking levels and (B) repeatability of the extraction procedure calculated as the relative standard deviation. Data for all tested sample preparation workflows, i.e., protein precipitation (PPT) in tubes and 96-well plates, phospholipid removal (PLR) in 96-well plates, are presented for plasma (top panel) and urine (bottom panel).

### Precision

The precision in terms of extraction repeatability was assessed as the RSD of extraction recoveries of QC samples (pre-extraction spiked) (Figure 2B). An RSD of <30% was considered acceptable. In plasma, >95% of compounds passed the acceptance criteria for both spiking levels in the PPT-tube-based protocol. For the plate-based workflow, 97% of both spiking levels showed acceptable RSDs. For PLR, only 9% in the high, but 94% in the low-spiking level resulted in RSDs of <30%. In urine, the PPT-tube-based workflow resulted in 99% and 86% of RSDs <30% in high- and low-spiking levels. The PPT-plate-based workflow had acceptable RSDs for 80% and 35% of compounds. The median RSD of the PLR workflow was highest for the higher spiking level (35%). Worse repeatability of the PLR workflow is also reflected in the lower number of compounds passing the cutoff of RSD <30% (28% for the high-spiking level and 99% for the low-spiking level). Although both PPT workflows show similar performance and acceptable variability compared with the PLR workflow, the PPT-tube-based approach shows superior RSDs, which might be caused by more rigorous vortexing. Calculated RSD values for each workflow and matrix are reported in Table S4 for plasma and Table S5 for urine.

### Characterization of Biological Samples

Due to the high sensitivity of the LC–HRMS system, background signals in pooled samples used in spiking experiments are common and, consequently, were examined in detail. In total, 27 compounds out of the targeted panel were identified in the nonspiked heparin plasma, including methylparaben, bisphenol A, acetaminophen, and perfluorononanoate. In the pooled urine sample, 20 compounds were identified, including enterolactone, kaempferol, and genistein. Calculated concentrations for each compound and workflow can be found in Table S7.

### Background Contamination in the Tested Sample Preparation Protocols

The presence of background contamination for each workflow was evaluated using process blanks. This is particularly relevant in exposomics studies since many of the target compounds can be present in labware, lab dust, or LC–MS parts. Background contamination was determined for eight analytes (dibutyl phthalate, *n*-butylbenzenesulfonamide, tri-*n*-butyl phosphate, triphenyl phosphate, perfluorobutanesulfonic acid, tris­(2-chlorethyl) phosphate, diethyl phthalate, and *p*-nitrophenol), regardless of the sample matrix and sample preparation method. Further details on background contamination can be found in the Supporting Information and Table S8.

### Applicability for Nontargeted Metabolomics/Exposomics

To investigate method performance beyond the target analytes, applicability for NTA and suspect screening applications was tested, which is more challenging than characterization of targeted (multianalyte) assays. The total number of features, annotated and identified analytes, and fold changes (FC > 2) of relative area under curve values of annotated features between methods were used to compare method performance (Figure 3). To allow for comparability, area-under-curve values (AUCs) obtained after DNS were corrected by the dilution factor. For both matrices, PPT in plates yielded the highest number of features after blank- and QC-based filtering. In plasma samples, 17 exposure compounds were identified (confidence level of 1) with all three tested workflows. These include cotinine, trans-3-hydroxycotinine, ibuprofen, ibuprofen glucuronide, perfluorooctanesulfonic acid, and perfluorooctanoic acid. The lowest number of identified analytes was observed for DNS and PLR in urine, highlighting the lower overall sensitivity of DNS. For PLR, several identified compounds were filtered out due to limited repeatability (i.e., RSD > 30%). In urine, compounds such as kaempferol, genistein, and enterolactone were identified and >190 others annotated. A full list of annotated features, including various identifiers and chemical classes can be found in Tables S9 and S10. A visual overview of the identified chemical classes is presented in Figure S2.

**Figure 3:**
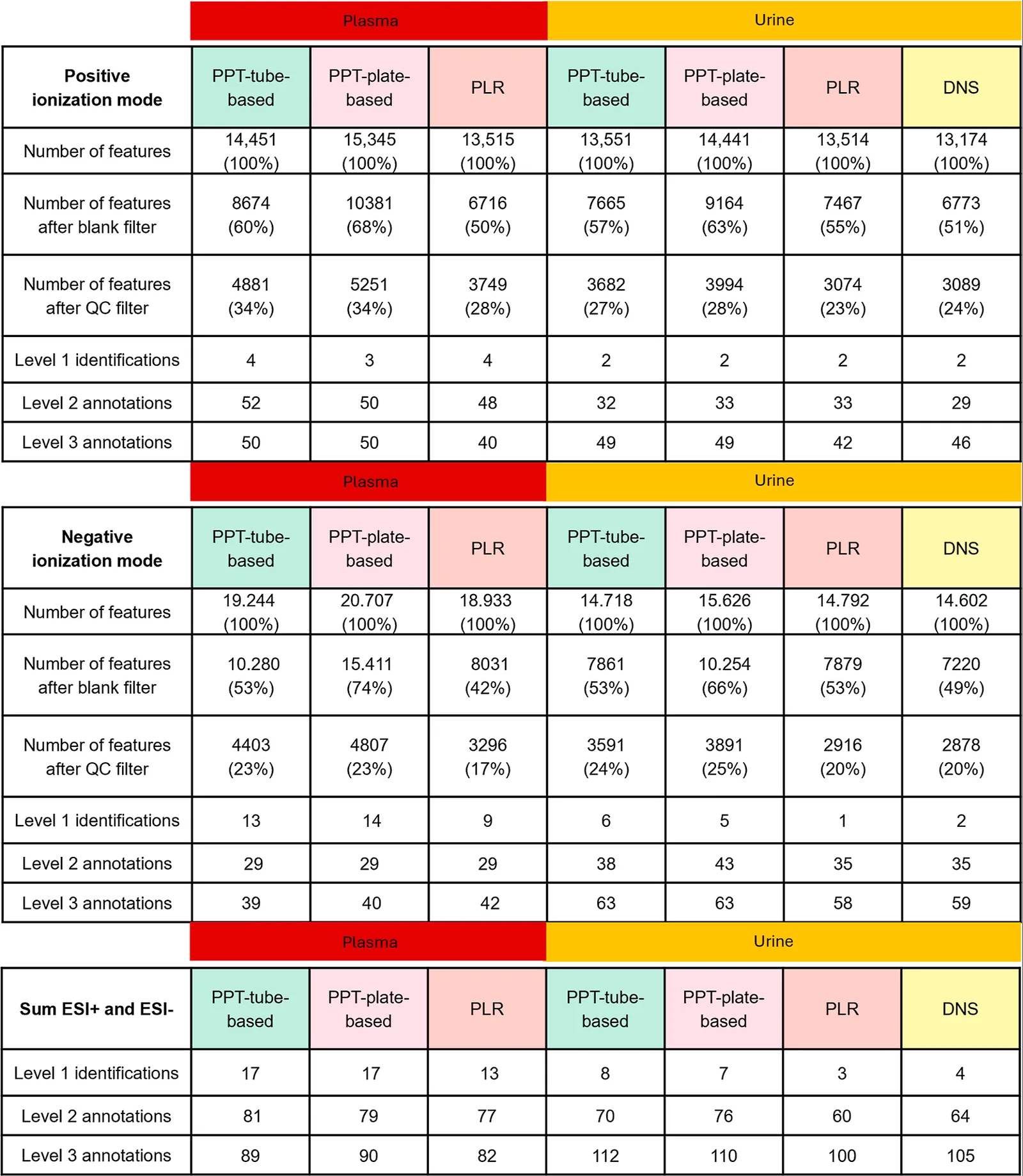
Nontargeted evaluation of different sample preparation protocols. Total number of features, level 1 identifications and level 2–3 annotations measured by LC–HRMS stratified by polarity, sample preparation approach, and biological matrix.

Comparison of feature AUCs using the tube- and plate-based PPT showed that >70% of annotated features in urine and plasma were not substantially different (FC > 2, Figures S3–S5, Table S11). In plasma, AUCs for 29% of annotations were decreased, and 15% were elevated using the PLR workflow. The urine DNS approach resulted in lower AUCs for 24% of all annotated features. The PLR workflow resulted in higher AUCs for 24% of the annotated features and decreased AUCs for another 23% of annotations. Corresponding fold changes are provided in detail in Table S11. Examples for extracted ion chromatograms (EICs) for annotated features in urine and their MS2 matches to MoNA can be found in Figure 4A–C. Further examples for EICs of annotations in plasma, some of which have been previously identified in prospective nested case–control metabolomics studies of breast cancer,^26^ can be found in Figure S6A–C. Interestingly, we observed changing chromatographic separation performance depending on the workflow for several analytes. For instance, kynurenic acid (level 3 annotation) resulted in a single peak in urine data after using DNS or PPT, whereas two distinct chromatographic peaks were observed after PLR treatment (Figure 4C). Phospholipids are major constituents of cell membranes. Their presence has an effect on analytical method performance,^27^ and differences in chromatographic behavior might be related to different compositions of the sample extracts.

**Figure 4:**
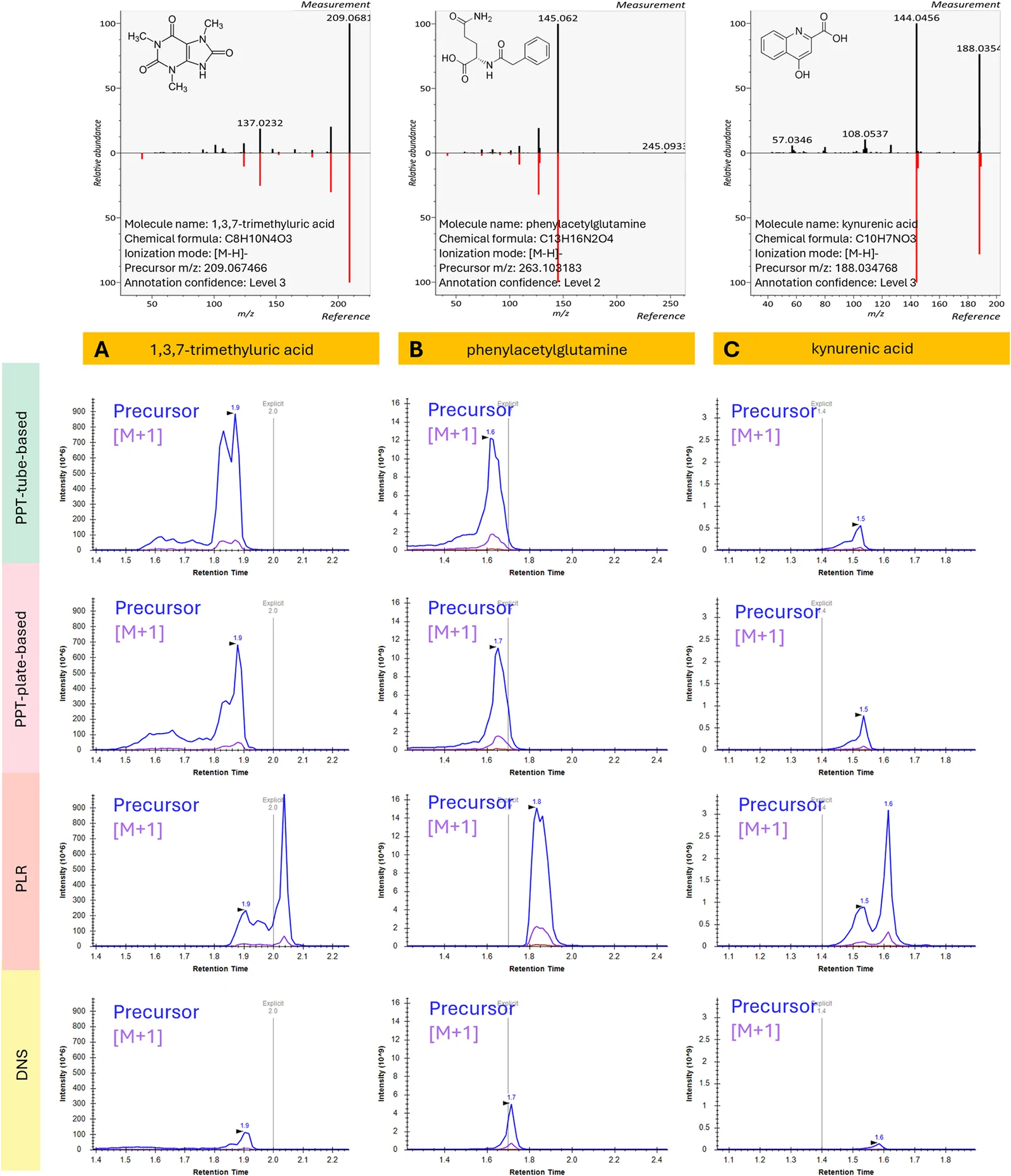
EICs and MS2 matches to reference spectra for selected features in pooled nonspiked urine. Data are presented for the four tested sample preparation protocols (protein precipitation (PPT) in tubes versus 96-well plates, PPT and phospholipid removal in 96-well plates (PLR), and dilute-and-shoot (DNS). Differences in chromatographic behavior for certain analytes such as kynurenic acid (C) were observed between PLR and other workflows.

### Practical Implications for Large-Scale Epidemiological Applications

To assess the potential of the tested protocols for high-throughput applications, time requirements for the preparation of 96 and 1000 biological samples were estimated, comparing the PPT-tube-based to the plate-based workflows. Manual (i.e., active) laboratory work for the tube-based workflow was estimated to result in >132 h for 1000 samples, while the plate-based workflow requires approximately 26 h. Thus, the plate-based workflow was estimated to be about five times more efficient. A detailed description of the estimated time requirements is presented in the Supporting Information and Table S12.

### Application to Certified Reference Material NIST SRM1950

Since the PPT-plate-based workflow offered the best compromise between performance and time- and cost-efficiency, it was applied to characterize exposure chemicals in the NIST SRM1950 certified reference material. Concentrations of detected compounds were compared to reference values, where applicable. In total, 22 analytes were detected, and a (semi-)­quantitative concentration value was calculated for 13 (Figure 5). Calculated concentrations were compared to available certified reference concentrations for five PFAS. In addition, data was compared to results from a previously published study for the same SRM material using a different analytical workflow.^11^ Reported (semi-)­quantitative concentrations of compounds detected using the PPT-plate-based workflow were mostly similar to reference values, especially for PFAS compounds. For example, herein, a concentration of 0.17 ± 0.02 ng/mL was reported for perfluoroundecanoic acid, while the NIST reference value is 0.186 ± 0.003 ng/mL. Additionally, we report 0.24 ± 0.03 ng/mL for perfluorodecanoic acid, while NIST reports 0.322 ± 0.007 ng/mL. Considerable differences in concentrations were observed for perfluorooctanesulfonic acid (PFOS), with the established protocol overestimating concentrations compared to the reference values (10.64 ± 0.13 ng/mL), indicating that the PPT-plate-based workflow is not suitable for the accurate quantification of this compound without further dilution. Since the peak area of PFOS in the sample exceeded the highest point in the calibration curve, this is a possible explanation why quantification was not as accurate as for the analytes within the verified concentration range. For certain analytes such as cotinine, trans-3-hydroxycotinine, ibuprofen, or acetaminophen, the commercial plasma used showed high background levels, rendering quantification and/or calculation of extraction recoveries nonfeasible, since the added concentrations of the compound in the spiking solution were too low compared to the high background signals and therefore indistinguishable. In these cases, the highest spiked concentration was stated because concentrations were outside of the analytical range of the used method. Furthermore, metribuzin and propylparaben were detected in the NIST SRM1950 with concentrations below the lowest spiking level in the matrix-matched calibration curve (0.1 ng/mL and 0.2 ng/mL, respectively) and were therefore labeled as <LLOQ. *N*-Butylbenzenesulfonamide (3.1 ± 2.4 ng/mL) was reported with a >10-fold higher concentration by Verri Hernandes and Warth 2024 (44 ng/mL)^11^ compared to this study. Six new compounds were determined in this study, i.e., ibuprofen and its glucuronide, tramadol, as well as mono butyl phthalate, 2-naphthol, and perfluorobutanesulfonic acid. Thus, no suitable reference concentration was available. Reported concentrations, as well as EICs for ibuprofen, propylparaben, PFOS, and tramadol in the NIST SRM1950, the highest spiking level in matrix and a process blank are depicted in Figure 5.

**Figure 5:**
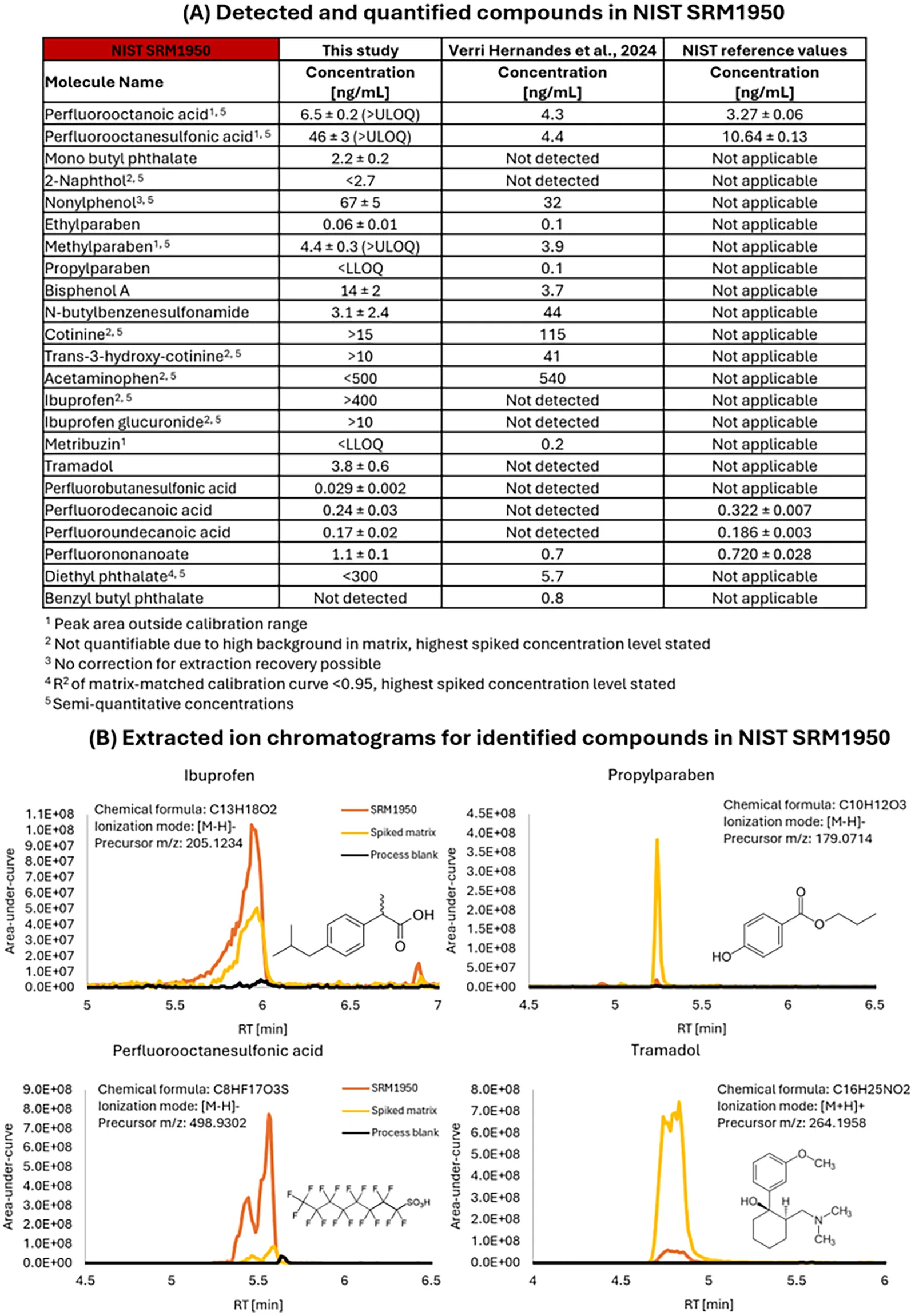
Application of the protein precipitation plate-based approach to a certified reference material (NIST SRM1950). (A) Calculated concentration of identified analytes in comparison to previously published data and certified reference values. Analytes where extraction recovery could not be assessed, calibration curve showed insufficient data points (<5) or where the peak area outside the calibration curve range were classified as semi-quantitative. (B) EICs of four detected compounds and comparison to process blanks and a spiked matrix sample.

### Limitations

Despite demonstrating the capability of the PPT-plate-based workflow for comprehensive high-throughput omics mass spectrometry, several limitations remain. The exposure compounds used to evaluate workflow performance were selected to cover multiple chemical classes, origins of exposures, and routes. Nevertheless, expansion of the panel is warranted to holistically characterize the exposome. Notably, exposomics assays need to cover extremely diverse analytes, and typically, a balance between analyte coverage and analytical figures of merit is required.^20^ Thus, a balanced method enables the quantification of key analytes while still allowing for (semi-)­quantification and screening of a more diverse set of exposure compounds. Despite these limitations, such methods may represent a suitable compromise to characterize the exposome without claiming analytical perfection, which may not be required for this purpose.^28^ Although the established sample preparation approach substantially improves sample throughput, automated sample and liquid handling systems would further improve the throughput and repeatability. The presented version of the protein precipitation workflow in plates already offers a suitable method performance for most analytes. Yet, additional optimization of the mixing steps using plate shakers might further improve the overall method performance.

## Conclusion and Outlook

Three high-throughput alternatives to commonly employed tube-based sample preparation protocols for human urine and two alternatives for plasma were tested, and their feasibility for combined exposomics/metabolomics applications was assessed. Workflows were compared based on extraction recovery and precision (RSD) for a panel of more than 200 exposure compounds, as well as applicability for nontargeted analysis. The transfer of a tube-based protein precipitation workflow to 96-well plates resulted in a comparable method performance for both matrices. Furthermore, the workflow was successfully applied to a reference material (SRM1950), resulting in comparable concentrations and numbers of detected toxicants. Future work includes application of this workflow to a case–control study of breast cancer nested in a prospective cohort including measurements of 1000+ biological samples for identification of biomarkers of susceptibility.

## Supplementary Material





## Data Availability

Raw data have been deposited in the MetaboLights^29^ repository with the study identifier MTBLS15100.

## Notes

Detailed information on the method, including compound list, concentration levels, method evaluation parameters, and annotation tables (XLSX)
Detailed information on materials, experimental laboratory work, as well as additional results (PDF)
