# Supplementary material for: Fast and Scalable Sample Preparation for Integrated Metabolome and Exposome-Wide Association Studies (MWAS/ExWAS)
Source: Anal Chem. 2026 Aug 23;98(35):26044–53. doi: 10.1021/acs.analchem.6c03820 (PMC13564032; doi:10.1021/acs.analchem.6c03820)
Supplement: Supplementary file 1 [file ac6c03820_si_002.pdf]

Supporting Information

Fast and Scalable Sample Preparation for Integrated Metabolome and Exposome-Wide Association Studies (MWAS/ExWAS)

Julia Füreder[a,b], Amira Müller[a], Giulia Guerra[a,c], Sabina Sieri[c], Max Lennart Feuerstein[a,d], and Benedikt Warth[a,b,d,*]

[a]University of Vienna, Faculty of Chemistry, Department of Food Chemistry and Toxicology, Währinger Straße 38, 1090 Vienna, Austria

[b]University of Vienna, Faculty of Chemistry, Vienna Doctoral School in Chemistry, Währinger Straße 38-42, 1090, Vienna, Austria

[c]Epidemiology and Prevention Unit, Fondazione IRCCS Istituto Nazionale dei Tumori, 20133 Milan, Italy

[d]Exposome Austria, National Research Infrastructure and EIRENE-AT Node, Austria

*Corresponding author: Benedikt Warth, benedikt.warth@univie.ac.at, +43-1-4277-70806.

Table of contents

Materials. 2

Sample preparation. 2

- Benchmarking tube-based protein precipitation workflow. 2
- 96-well plate-based protein precipitation (and phospholipid removal) approaches. 2
- Dilute-and-shoot approach. 2

Data Acquisition. 2

- Measurements of NIST SRM1950 2
- Quality control protocol. 3

Data Analysis. 3

- Nontargeted metabolomics/exposomics. 3

Results and Discussion. 3

- Linearity of matrix-matched calibration curves. 3
- Background contamination in the different sample preparation workflows. 4
- Applicability of workflows for nontargeted metabolomics applications. 4
- Practical implications for high-throughput applications. 8

References 9

Materials.

LC-MS grade water was purchased from VWR chemicals (Radnor, Pennsylvania, United States). Methanol (MeOH) and acetonitrile (ACN) were purchased in LC-MS grade from Honeywell (Charlotte, North Carolina, United States) and Fisher Scientific (Waltham, Massachusetts, United States). Ammonium fluoride (NH_4F) was purchased from Honeywell (Charlotte, North Carolina, United States). Polypropylene reaction tubes (1.5 mL) were purchased from Sarstedt (Nürnberg, Germany). ISOLUTE PPT+ protein precipitation plates, as well as ISOLUTE PLD+ protein and phospholipid removal plates were purchased from Biotage (Uppsala, Sweden).

Sample preparation.

Benchmarking tube-based protein precipitation workflow.

The sample preparation procedure used for benchmarking is a simple liquid extraction and protein precipitation workflow, commonly employed for exposomics/metabolomics assays in our laboratory. The method was previously validated and offers a broad analyte coverage[1]. During the whole sample preparation procedure, the samples were kept on ice. A volume of 100 µL sample (plasma or urine) was mixed with 400 µL of extraction solvent (ACN/MeOH (1:1, v/v)) in 1.5 mL polypropylene tubes. For the pre-extraction-spiked samples, 300 µL of extraction solvent was mixed with 100 µL of the respective standard mix prepared in extraction solvent. Samples were vortexed and sonicated on ice (10 min). Proteins were precipitated by placing the samples at -20°C overnight. Tubes were centrifuged at 18,000 rcf and 4°C (10 min). A volume of 350 µL of the supernatant was transferred to fresh tubes and evaporated to dryness at 7°C in a vacuum concentrator (CentriVap, Labconco, Kansas City, Missouri, United States) and stored at -20°C until resuspension. The residues were reconstituted in 70 µL of reconstitution solvent (ACN/water (1:1, v/v)) followed by vortexing. Matrix-matched calibration samples were reconstituted in 70 µL of the standard mix in the respective concentration level prepared in ACN/water (1:1, v/v). Samples were sonicated on ice (10 min) and centrifuged at 18,000 rcf and 4°C (10 min). Supernatants were transferred to LC vials and measured directly (plasma) or stored at -20°C until analysis (urine).

96-well plate-based protein precipitation (and phospholipid removal) approaches.

Two high-throughput alternatives to the tube-based protein precipitation benchmarking procedure described above were tested. They employed a protein removal (PPT-plate-based) or a protein and phospholipid removal filter plate (PLR). The sample preparation procedure for both plates was identical. During the whole procedure, the samples were kept on ice. A volume of 100 µL sample was mixed with 400 µL of extraction solvent (ACN/MeOH (1:1, v/v)) in the 96-well plate using a pipette. For the pre-extraction-spiked samples, 300 µL of extraction solvent was used and 100 µL of the respective standard mix prepared in extraction solvent. Proteins were precipitated by storing the plate at -20°C overnight and centrifugation was performed at 1000 rcf and 4°C (15 min) to collect the supernatant. Then, the samples were evaporated to dryness in a vacuum concentrator at 7°C (CentriVap, Labconco, Kansas City, Missouri, United States) and stored at -20°C until resuspension. The residues were reconstituted in 100 µL of reconstitution solvent (ACN/water (1:1, v/v)) and mixed on a plate shaker (Incu-Mixer MP4, Benchmark Scientific, Sayreville, New Jersey, United States) at 1000 rpm and room temperature (10 min). Matrix-matched calibration samples were reconstituted in 100 µL of the standard mix in the respective concentration level prepared in ACN/water (1:1, v/v). Finally, the extracts were transferred to LC vials and measured directly (plasma) or stored at -20°C until analysis (urine).

Dilute-and-shoot approach.

Urine characteristically possesses a lower protein content compared to plasma; consequently less labour-intensive dilution of the sample is often feasible and commonly used in mass spectrometry-based assays[2]. Therefore, a dilute-and-shoot approach was evaluated as another high-throughput alternative for urine samples. During the whole procedure, the samples were kept on ice. A volume of 25 µL of urine sample was mixed with 100 µL of ACN/water (1:1, v/v). For matrix-matched calibration, 25 µL of urine sample were mixed with 100 µL of the standard mix in the respective concentration level prepared in ACN/water (1:1, v/v). Samples were vortexed and centrifuged at 18,000 rcf and 4°C (10 min). The supernatant was transferred to LC vials and stored at -20°C until analysis.

Data Acquisition.

Measurements of NIST SRM1950.

NIST SRM1950 samples were measured on the same instrument configuration used for the main experiment with slight modifications. Mobile phase B was composed of 95% (v/v) ACN with 5% water. The gradient was prolonged to a total runtime of 20 min by extending the equilibration step from 3 min to 8 min. The resolution was set to 60,000 at *m/z* 200 in full scan mode. A matrix-matched calibration curve was measured within the same run, using the same eluents, but the

methods from the main experiment. Quantification of the SRM1950 was done based on the matrix-matched calibration curve measured within the same run, but using the reported extraction recoveries from the main experiment.

Quality control protocol.

Several types of quality control (QC) samples were used during the sequences to ensure adequate data quality. A system suitability test (SST) was performed before each analytical sequence to ensure acceptable instrument performance. Details on the SST, such as LC-MS specific parameters as well as targeted analytes, can be found in **Table S2**. Solvent and process blanks were prepared and injected multiple times throughout the sequence to account for background contamination from the sample preparation process or the measurement. Furthermore, the multi-analyte mixture in neat solvent and matrix was injected multiple times throughout the sequence at different concentration levels (six concentration levels for matrix-matched calibration curve and seven concentration levels for solvent calibration curve). To furthermore estimate instrument variability and signal drift, a spiked QC from the PLR plate was re-injected three times at the end of the sequence. The data acquisition order for the LC-HRMS measurements is illustrated in **Figure 1C**.

Data Analysis.

Nontargeted metabolomics/exposomics.

Raw full scan data files were separated into positive and negative polarity and converted to mzML format, including the vendor-specific peak picking algorithm using ProteoWizard's msConvert. Generated full scan and MS2 fragmentation spectra were analyzed using MS-DIAL (4.9.221218). An intensity cutoff of 100,000 counts was used. A retention time tolerance of 0.3 min and 0.015 Da for MS1 spectra was used to align data. MassBank of North America (MoNA, https://mona.fiehnlab.ucdavis.edu/) was used for annotation of spectral matches. The classification of annotations was done based on level of condifence[3]. "Level 1" describes a match confirmed by authentic reference standards, "Level 2" confirmation by a spectral library match, and "Level 3" confirmation by spectral library match, but with the possibility of isomeric structures. Lower confidence levels ("Level 4" and "Level 5") were not considered for this evaluation. For Level 1 identifications, a retention time tolerance of 0.3 min compared to the spiked matrix was set. Further data processing was done using R Studio (version 2022.12.0+353), MetaboAnalyst (version 6.0) and Microsoft Office 365. Peak areas of the matrix samples from the dilute-and-shoot approach were corrected for dilution by multiplying with the dilution factor. Exported feature tables were blank filtered (average feature area in sample must be higher than three times average feature area in process blanks) and QC filtered (only features with RSD <30% were kept). Only annotations with a mass error below 5 ppm were accepted. Annotation tables from positive and negative ionization modes were merged and for annotations with identical chemical formulas that were annotated in both polarities ([M+H]+ and [M-H]-) or different adduct forms (e.g. [M+H-H2O]+, [M+Na]+) within a retention time window of 0.25 min, only the feature with higher intensity was kept for statistical analysis. Peak intensities were log-transformed (base 10) using MetaboAnalyst (version 6.0) and auto-scaling was applied.

Results and Discussion.

Linearity of matrix-matched calibration curves.

Most matrix-matched calibration curves showed acceptable linearity for each workflow and matrix. For plasma, 69% of all compounds for the PPT-tube-based workflow passed the filtering criteria of at least two calibration points and had an R^2>0.95 with a median R^2 of 0.997. For the plate-based workflow, 70% of calibration curves were acceptable with a median R^2 of 0.999, while for the PLR workflow, 71% of compounds passed the criteria with a median R^2 of 0.997. Calculation of median R^2 was performed only for calibration curves with >2 calibration points. In urine, 68% of all compounds for the PPT-tube-based workflow passed the criteria with a median R^2 of 0.996, 69% for the plate-based workflow with a median R^2 of 0.999, 67% and a median R^2 of 0.996 for the PLR workflow and 72% and a median R^2 of 0.975 for the DNS. An example for the matrix-matched calibration curves for zearalenone, a commonly found mycotoxin in human urine, for all four urine workflows can be found in **Figure S1**. The concentration levels on the x-Axis are arbitrary calibration levels. Level 100 corresponds to a concentration of 3 ng/mL of zearalenone. Real concentration levels can be found in **Table S1**. DNS workflow shows the steepest slope, followed by the PLR, plate-based and tube-based protein precipitation workflow. The variation in the steepness of the slope likely reflects differences in matrix effect across the different extraction methods. Notably, there is also a difference in slope and the lowest spiking level within the linear range of the calibration curve (LLOQ) between both PPT approaches (plate- versus tube-based), which might be due to the introduction of an additional filtering material for the plate-based approach that could influence the removal of matrix compounds.

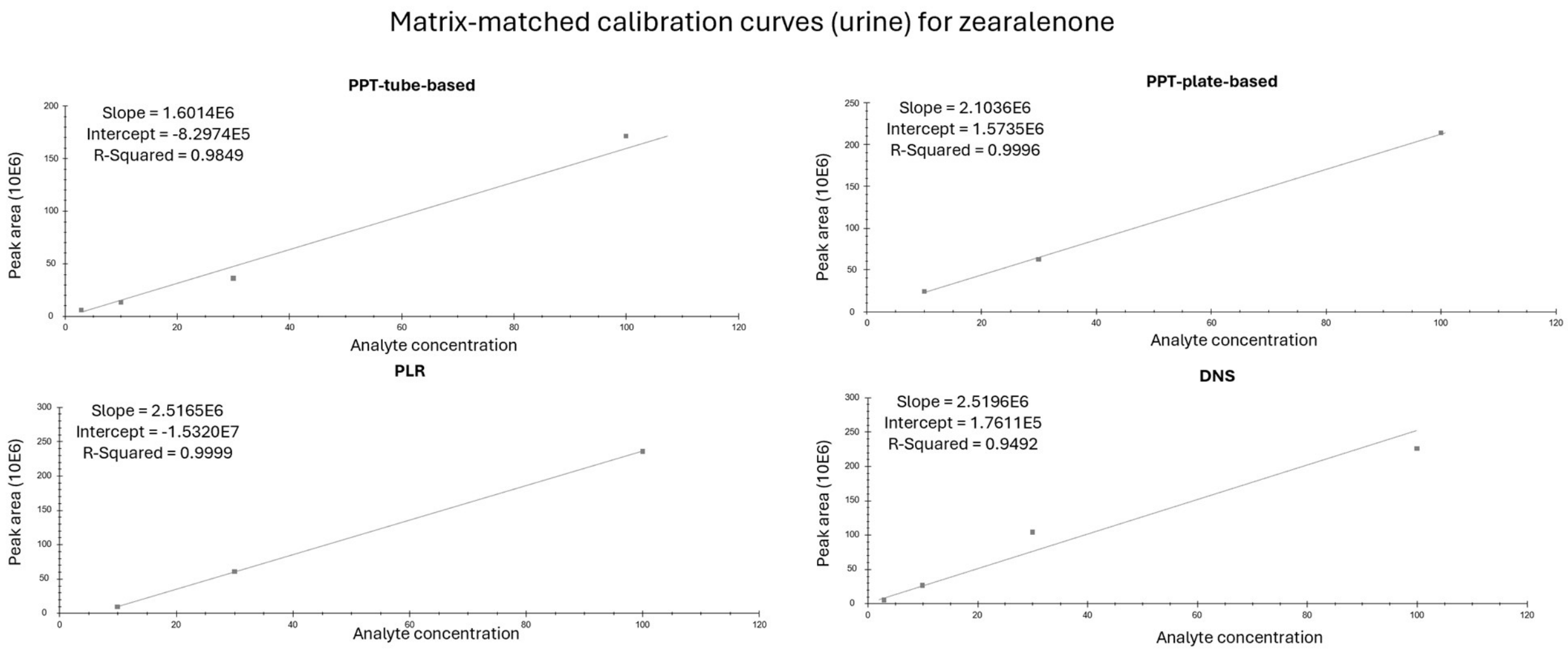

Figure S1: Matrix-matched calibration curves for all urine workflows of zearalenone, a commonly found mycotoxin in urine.

Background contamination in the different sample preparation workflows.

In addition to the eight universally detected compounds in the process blanks described in the main text, nonylphenol and bisphenol A were detected in all but the DNS workflow. The PLR plate was additionally contaminated with mono-iso-nonyl phthalate, bisphenol E and bisphenol F. 4-hydroxybenzophenone was only detected in the process blanks of plasma. Detected background contaminations can be found in **Table S8**. Most compounds showed constant background levels in all process blank replicates, however, for bisphenol A and 2-naphthol, some unique samples showed much higher concentrations (e.g. >5 times higher). Some of these compounds have been reported before as background contamination, including bisphenol A, bisphenol F (about 10-fold lower concentration previously reported from solvents than found in the process blanks from the PLR plates herein), dibutylphthalate, n-butylbenzenesulfonamide, tri-n-butylphosphate and triphenylphosphate[4].

Applicability of workflows for nontargeted metabolomics applications.

During nontargeted data evaluation, a total of 208 compounds were annotated or identified in plasma and 203 in urine by spectral matching to MoNA. The full annotation table is available in **Table S9 and S10**. A visual overview of chemical classes (according to HMDB.ca) of these identifications and annotations is presented in **Figure S2**. For some compounds, no information on chemical class was available and they were therefore labeled as “other molecules”.

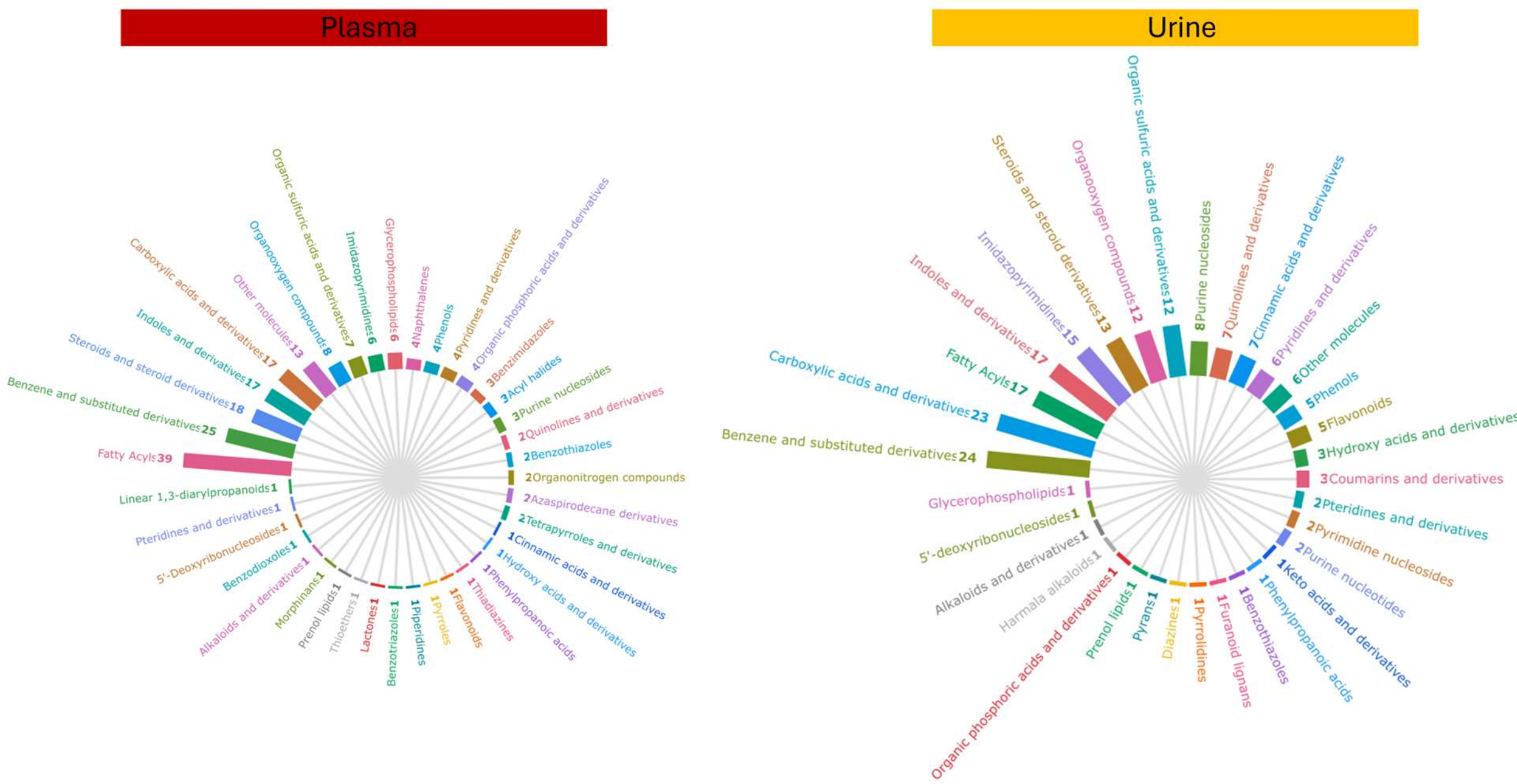

Figure S2: Overview on number of annotated and identified compounds per chemical class, as described in Tables S9 and S10 for both matrices. Chemical class was derived from human metabolome database (HMDB.ca). A total of 208 compounds were annotated or identified in human plasma and 203 in human urine.

Fold change analysis with a fold-change threshold of 2 was applied to compare protocols at the annotation level. Visualization of the fold changes for all workflows and matrices can be found in **Figures S3-5**. Fold change values for the annotated features can be found in **Table S10**.

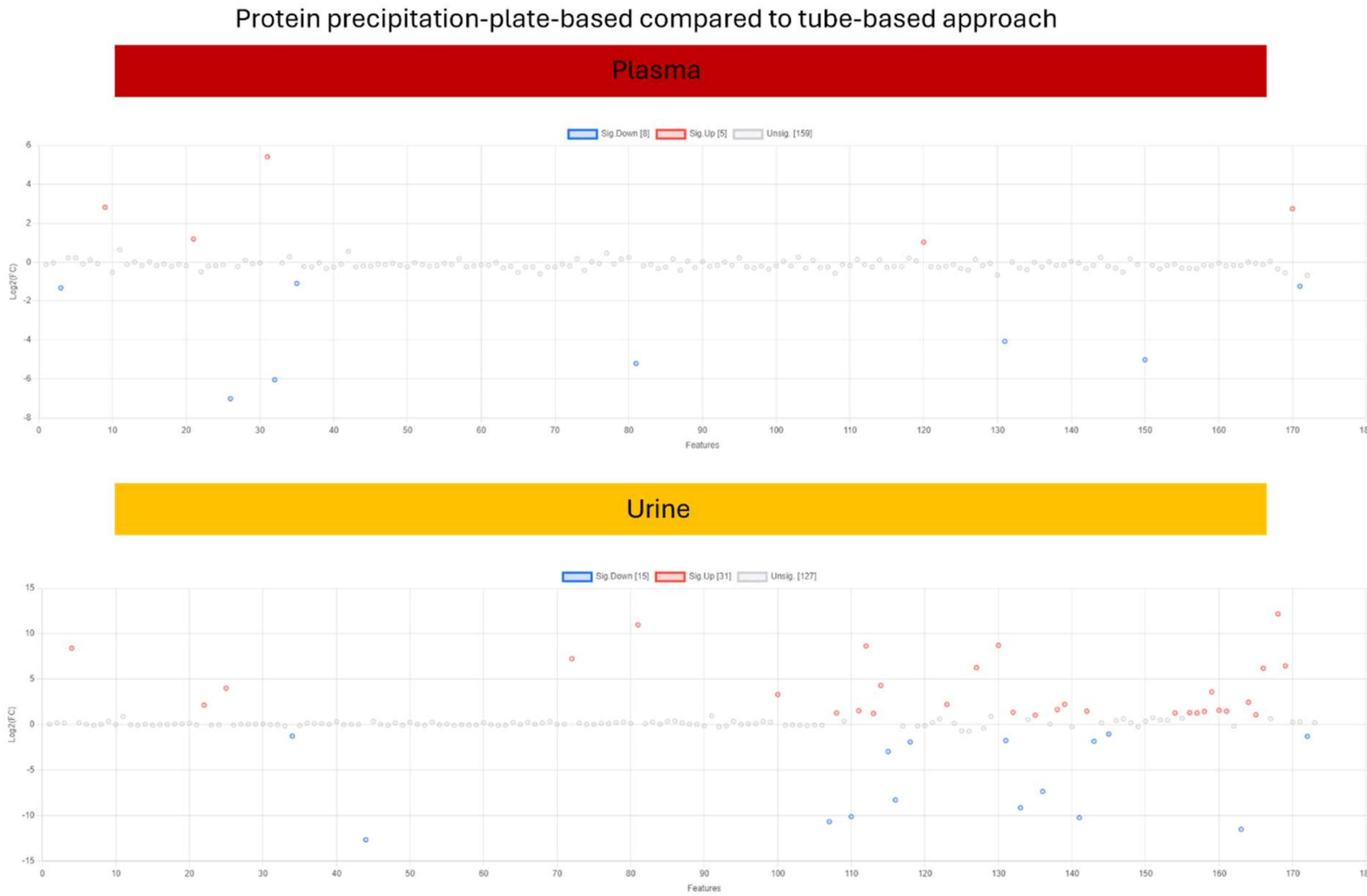

Figure S3: Fold change comparison of log-transformed area under curve values (AUC) of annotated features (confidence level 1-3) with a fold change threshold of 2. 96-well plate-based protein precipitation workflow was benchmarked against an established tube-based protein precipitation protocol[1,5] commonly used in metabolomics applications, resulting in similar performance with more than 70% of overlapping annotated features for both matrices.

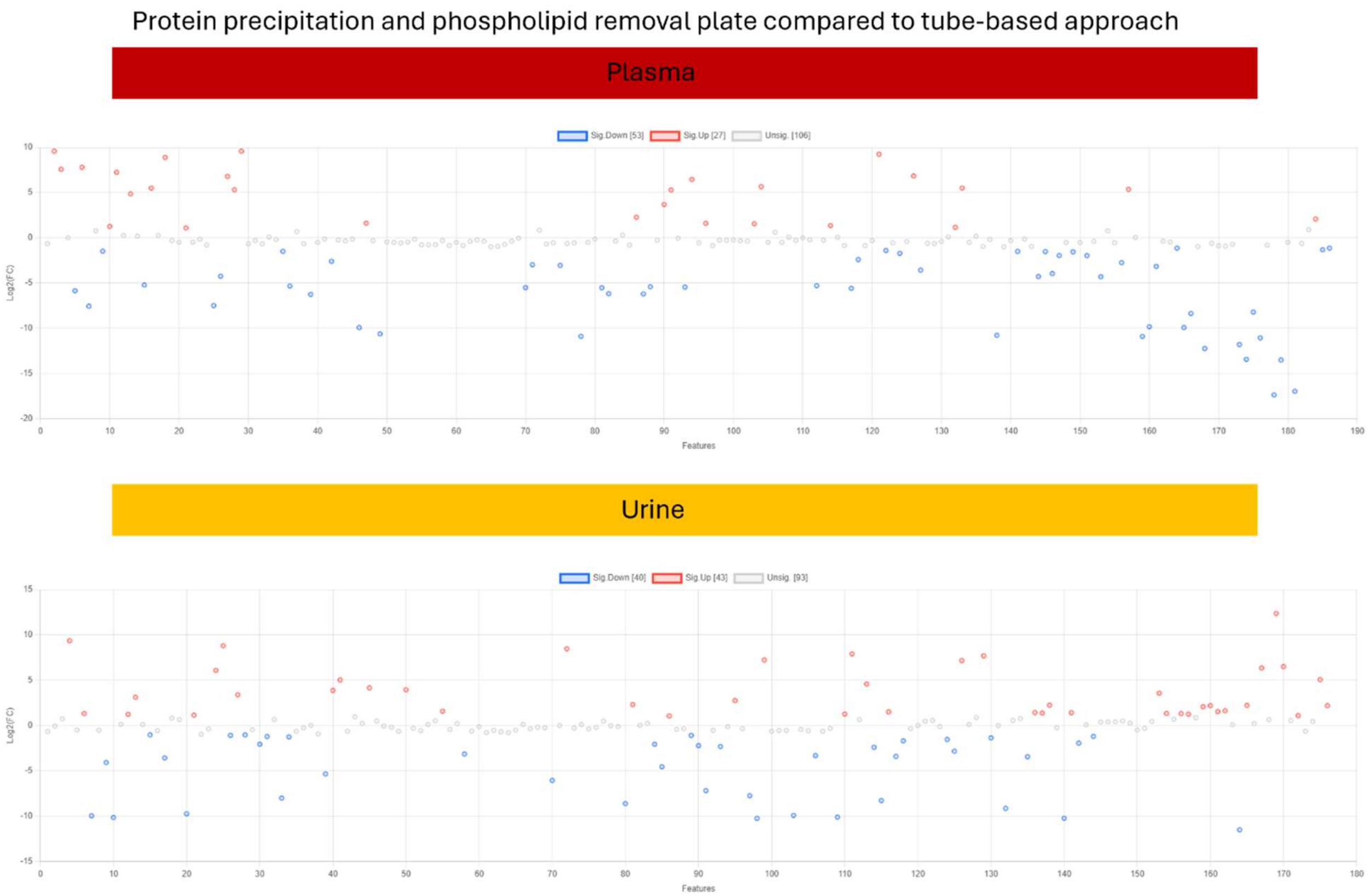

Figure S4: Fold change comparison of log-transformed area under curve values (AUC) of annotated features (confidence level 1-3) with a fold change threshold of 2. 96-well plate-based protein precipitation and phospholipid removal workflow was benchmarked against an established tube-based protein precipitation protocol[1,5] commonly used in metabolomics applications.

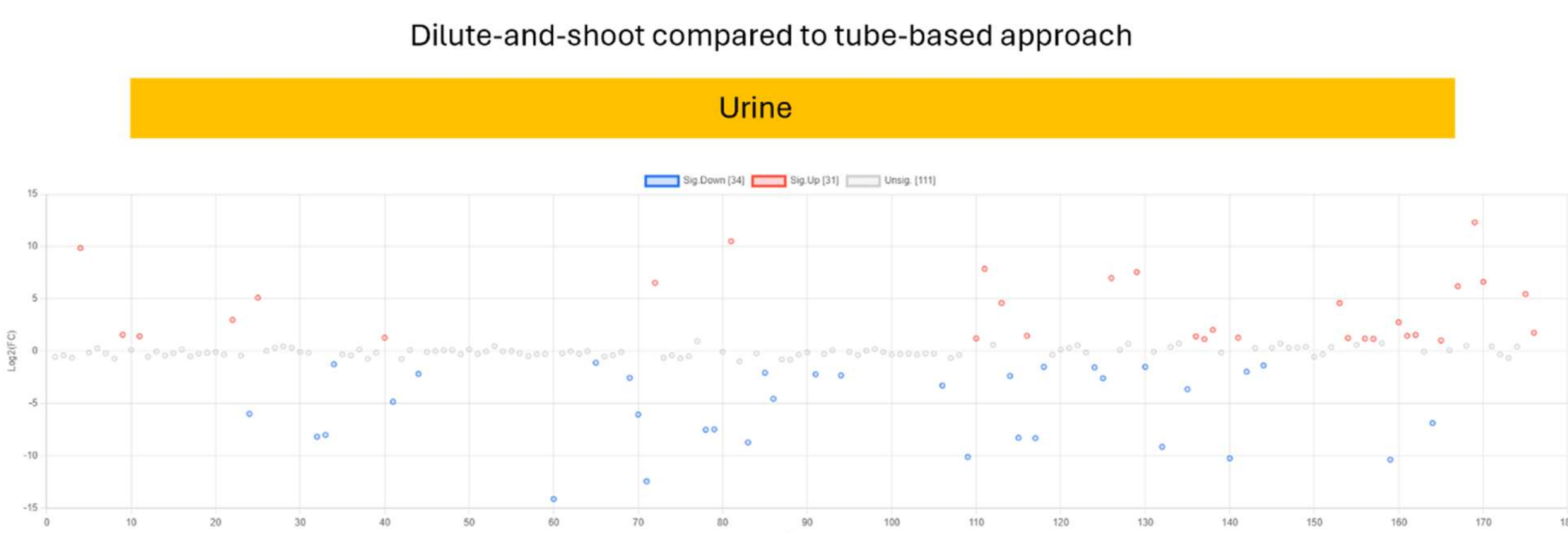

Figure S5: Fold change comparison of log-transformed area under curve values (AUC) of annotated features (confidence level 1-3) with a fold change threshold of 2. Dilute-and-shoot workflow in urine was benchmarked against an established tube-based protein precipitation protocol[1,5] commonly used in metabolomics applications.

Chaker et al., 2022, performed an evaluation of 12 sample preparation methods for chemical exposomics on blood and serum samples, including protein precipitation and protein precipitation and phospholipid removal protocols[6]. A positive log fold change for piperine was observed in samples prepared with the PLR protocol compared to the PPT workflow for both tested cohort plasma samples. In this study, piperine (level 3 annotation) also showed higher values in urine prepared with the PLR workflow than with the PPT-tube based benchmark protocol (fold change 4.6). Furthermore, cotinine showed higher values in their PPT protocol, while it's metabolite trans-3-hydroxycotinine was elevated using the PLR protocol. In this study, both

compounds had lower AUCs with the PLR than the PPT workflow in plasma (fold change 0.2 for cotinine and 0.3 for trans-3-hydroxycotinin). In a later study published by Chaker et al., specifically comparing chemical coverage in large-scale LC-HRMS applications, they show that certain chemical classes, such as phthalates are predominantly detected using the PLR workflow, while others such as LPC-like molecules are favored by the PPT workflow[7]. In the study at hand, we also observed that LysoPC(0:0/18:0) (Level 3 annotation) in plasma showed lower AUCs in the PLR workflow (fold change 8E-06). In contrast, phthalates such as dicyclohexyl phthalate or mono-(2-ethyl-5-hydroxyhexyl) phthalate show fold changes of 0.005 and 0.007 respectively in this study. Results from NTA studies should be compared cautiously, since sample preparation, instrumentation, data evaluation software and objective of the study, e.g. chemical coverage versus high sensitivity, can have a great impact[6].

Examples for extracted ion chromatograms for annotated features in plasma, where some have been previously identified in major prospective nested case-control metabolomics studies of breast cancer[8], as well as their MS2 match to MassBank of North America can be found **in Figure S6A-C**.

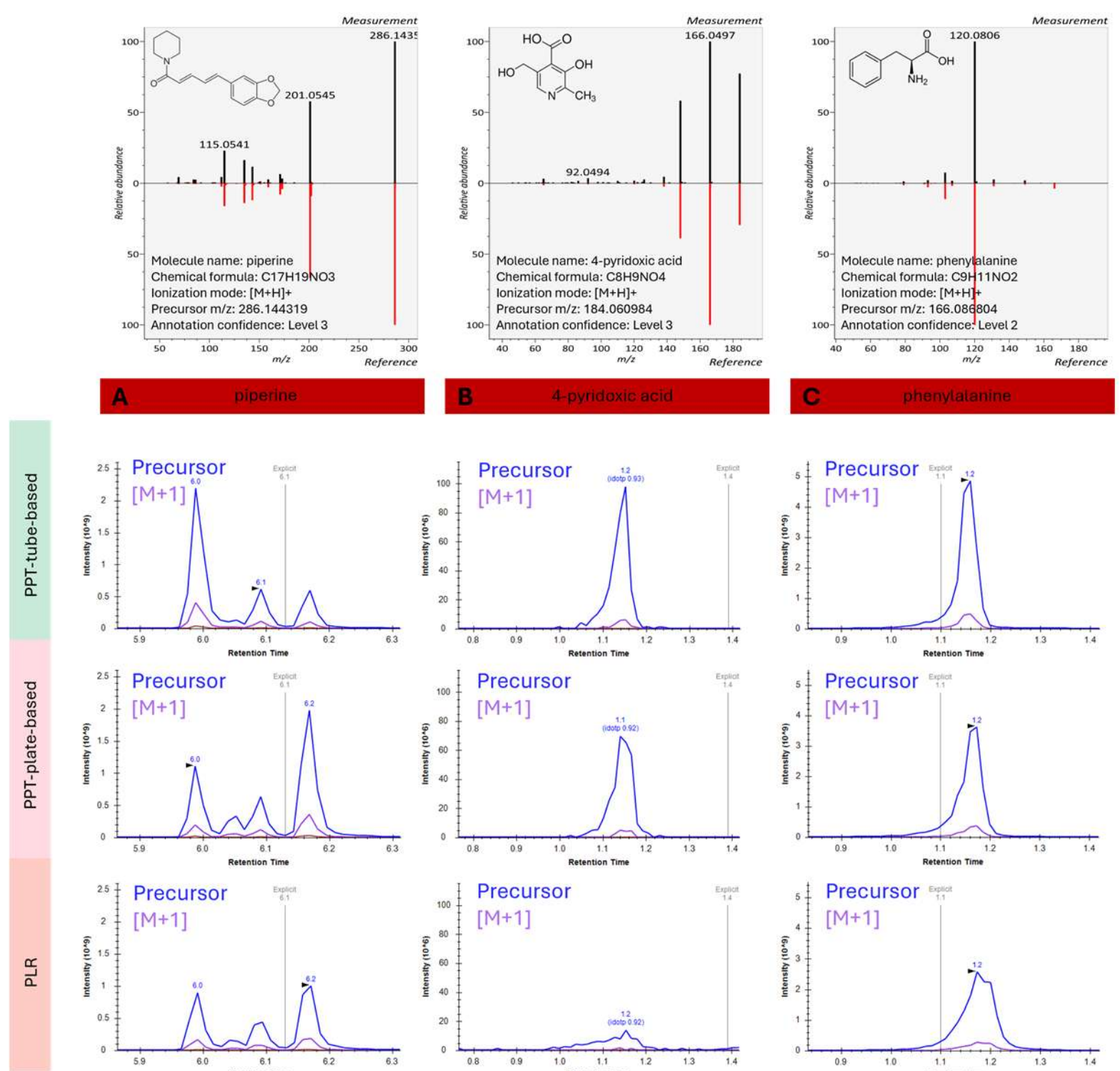

Figure S6: Extracted ion chromatograms, as well as MS2 matches of selected features in plasma, where some have been previously identified to be potential biomarkers of susceptibility for breast cancer[8], for all workflows (protein precipitation and phospholipid removal (PLR), and protein precipitation tube- and plate-based (PPT-tube-based and PPT-plate-based)).

Practical implications for high-throughput applications.

To assess the potential of the workflows for high-throughput applications, estimated time requirements for the preparation of 96 (equal to one plate) and 1000 biological samples were made, comparing the PPT-tube-based to the PPT-plate-based workflow. Active laboratory work for the tube-based workflow for 1000 biological samples is estimated to be more than 132 hours, while the plate-based workflow only requires about 26 hours. According to these estimations, the plate-based workflow is five times more efficient for the preparation of 1000 biological samples. Details of the time comparisons can be found in **Table S11**. Specifically, the manual labeling of the sample tubes is the most time-consuming step for the tube-based approach, assuming the use of an automated label printer and manual affixing of the printed label to the tubes and LC vials. Furthermore, the use of 96-well plates enables working with a (tip-spacing) multi-channel pipette for the transfer of the original sample to the plate, as well as the addition of any solvent (extraction or reconstitution solvent) in a more efficient manner. In this study, the sample and extraction solvent were mixed by pipetting up- and down ten times to assure optimal mixing. This could be streamlined in the future by using a plate shaker, as done for the reconstitution step. In this study, all

sample extracts, regardless of the sample preparation procedure, were transferred to LC vials, to not introduce additional contamination from 96-well injection plates and to keep the workflows as comparable as possible. For the preparation of 1000 samples it is assumed to use 96-well injection plates, saving additional time during labeling and transfer of extracts. The plate-based workflows as performed in this study already show improved throughput compared to the tube-based workflow and furthermore have the potential to be fully streamlined with the use of automated liquid handling systems.

References

1. Jamnik, T.; Flasch, M.; Braun, D.; Fareed, Y.; Wasinger, D.; Seki, D.; Berry, D.; Berger, A.; Wisgrill, L.; Warth, B. Next-generation biomonitoring of the early-life chemical exposome in neonatal and infant development. *Nature Communications* **2022**, *13*(1), 1–14.

2. Khamis, M. M.; Adamko, D. J.; El-Aneed, A. Mass spectrometric based approaches in urine metabolomics and biomarker discovery. *Mass Spectrometry Reviews* **2017**, *36*(2), 115–134.

3. Schymanski, E. L.; Jeon, J.; Gulde, R.; Fenner, K.; Ruff, M.; Singer, H. P.; Hollender, J. Identifying small molecules via high resolution mass spectrometry: Communicating confidence. *Environmental Science and Technology* **2014**, *48*(4), 2097–2098.

4. Hernandes, V. V.; Zeyda, M.; Wisgrill, L.; Warth, B. Dried blood spots analysis for targeted and non-targeted exposomics. *Environment International* **2025**, *205*.

5. Ivanisevic, J.; Zhu, Z. J.; Plate, L.; Tautenhahn, R.; Chen, S.; O'Brien, P. J.; Johnson, C. H.; Marletta, M. A.; Patti, G. J.; Siuzdak, G. Toward 'Omic scale metabolite profiling: A dual separation-mass spectrometry approach for coverage of lipid and central carbon metabolism. *Analytical Chemistry* **2013**, *85*(14), 6876–6884.

6. Chaker, J.; Kristensen, D. M.; Halldorsson, T. I.; Olsen, S. F.; Monfort, C.; Chevrier, C.; Jégou, B.; David, A. Comprehensive Evaluation of Blood Plasma and Serum Sample Preparations for HRMS-Based Chemical Exposomics: Overlaps and Specificities. *Analytical Chemistry* **2022**, *94*(2), 866–874.

7. Chaker, J.; Gorrochategui Matas, E.; Chevrier, C.; Campone, M.; Frenel, J. S.; Bigot, F.; Bessonneau, V.; David, A. Blood Sample Preparation for Human Chemical Exposomics: Insights from Large-Scale Applications. *Environmental Science and Technology* **2025**, *59*(40), 21421–21430.

8. Füreder, J.; Schernhammer, E. S.; Eliassen, A. H.; Sieri, S.; Warth, B. Metabolomics-enabled biomarker discovery in breast cancer research. *Trends in Endocrinology and Metabolism* **2026**, *37*(1), 68–82.
